# Evaluating the Hounsfield unit assignment and dose differences between CT‐based standard and deep learning‐based synthetic CT images for MRI‐only radiation therapy of the head and neck

**DOI:** 10.1002/acm2.14239

**Published:** 2023-12-21

**Authors:** Kamal Singhrao, Catherine Lu Dugan, Christina Calvin, Luis Pelayo, Sue Sun Yom, Jason Wing‐Hong Chan, Jessica Elizabeth Scholey, Lisa Singer

**Affiliations:** ^1^ Department of Radiation Oncology Brigham and Women's Hospital, Dana‐Farber Cancer Institute, Harvard Medical School Boston Massachusetts USA; ^2^ Department of Radiation Oncology University of California, San Francisco San Francisco California USA

**Keywords:** AI in radiation therapy, MRI‐only radiation therapy, synthetic CT

## Abstract

**Background:**

Magnetic resonance image only (MRI‐only) simulation for head and neck (H&N) radiotherapy (RT) could allow for single‐image modality planning with excellent soft tissue contrast. In the MRI‐only simulation workflow, synthetic computed tomography (sCT) is generated from MRI to provide electron density information for dose calculation. Bone/air regions produce little MRI signal which could lead to electron density misclassification in sCT. Establishing the dosimetric impact of this error could inform quality assurance (QA) procedures using MRI‐only RT planning or compensatory methods for accurate dosimetric calculation.

**Purpose:**

The aim of this study was to investigate if Hounsfield unit (HU) voxel misassignments from sCT images result in dosimetric errors in clinical treatment plans.

**Methods:**

Fourteen H&N cancer patients undergoing same‐day CT and 3T MRI simulation were retrospectively identified. MRI was deformed to the CT using multimodal deformable image registration. sCTs were generated from T1w DIXON MRIs using a commercially available deep learning‐based generator (MRIplanner, Spectronic Medical AB, Helsingborg, Sweden). Tissue voxel assignment was quantified by creating a CT‐derived HU threshold contour. CT/sCT HU differences for anatomical/target contours and tissue classification regions including air (<250 HU), adipose tissue (–250 HU to –51 HU), soft tissue (–50 HU to 199 HU), spongy (200 HU to 499 HU) and cortical bone (>500 HU) were quantified. t‐test was used to determine if sCT/CT HU differences were significant. The frequency of structures that had a HU difference > 80 HU (the CT window‐width setting for intra‐cranial structures) was computed to establish structure classification accuracy. Clinical intensity modulated radiation therapy (IMRT) treatment plans created on CT were retrospectively recalculated on sCT images and compared using the gamma metric.

**Results:**

The mean ratio of sCT HUs relative to CT for air, adipose tissue, soft tissue, spongy and cortical bone were 1.7 ± 0.3, 1.1 ± 0.1, 1.0 ± 0.1, 0.9 ± 0.1 and 0.8 ± 0.1 (value of 1 indicates perfect agreement). T‐tests (significance set at *t* = 0.05) identified differences in HU values for air, spongy and cortical bone in sCT images compared to CT. The structures with sCT/CT HU differences > 80 HU of note were the left and right (L/R) cochlea and mandible (>79% of the tested cohort), the oral cavity (for 57% of the tested cohort), the epiglottis (for 43% of the tested cohort) and the L/R TM joints (occurring > 29% of the cohort). In the case of the cochlea and TM joints, these structures contain dense bone/air interfaces. In the case of the oral cavity and mandible, these structures suffer the additional challenge of being positionally altered in CT versus MRI simulation (due to a non‐MR safe immobilizing bite block requiring absence of bite block in MR). Finally, the epiglottis HU assignment suffers from its small size and unstable positionality. Plans recalculated on sCT yielded global/local gamma pass rates of 95.5% ± 2% (3 mm, 3%) and 92.7% ± 2.1% (2 mm, 2%). The largest mean differences in D95, D_mean_, D50 dose volume histogram (DVH) metrics for organ‐at‐risk (OAR) and planning tumor volumes (PTVs) were 2.3% ± 3.0% and 0.7% ± 1.9% respectively.

**Conclusions:**

In this cohort, HU differences of CT and sCT were observed but did not translate into a reduction in gamma pass rates or differences in average PTV/OAR dose metrics greater than 3%. For sites such as the H&N where there are many tissue interfaces we did not observe large scale dose deviations but further studies using larger retrospective cohorts are merited to establish the variation in sCT dosimetric accuracy which could help to inform QA limits on clinical sCT usage.

## 1 INTRODUCTION

Magnetic resonance imaging (MRI) is increasingly used in head and neck (H&N) radiotherapy treatment (RT) planning.^1^, ^2^, ^3^, ^4^ MRI provides excellent visualization of soft tissue anatomy and more exact delineation of tumor extent, and thus augments anatomical information provided by computed tomography (CT) radiotherapy simulation imaging.^2^, ^5^, ^6^, ^7^, ^8^, ^9^, ^10^ MRI‐only simulation imaging for treatment planning could be advantageous for H&N sites due to excellent soft tissue contrast on a single modality image hence eliminating multimodality MRI/CT image registration errors which necessitate larger treatment margins.^11^, ^12^

As part of the MRI‐only workflow, synthetic computed tomography (sCT) images are produced from MRI simulation images to aid patient setup and provide electron density information.^13^, ^14^, ^15^ sCT images are generated from MRI because the latter does not provide electron density information.^11^, ^16^, ^17^ Accounting for tissue heterogeneity in external beam radiation therapy is important because it allows for planning systems to correct for photon fluence passing through tissues and more accurately map doses.^18^ sCT solutions developed for the H&N region include bulk density, statistical/machine learning, and deep learning methods.^19^, ^20^, ^21^, ^22^ The challenging and complex H&N anatomy, in particular its multiple air/bone interfaces and small bony structures, has meant that bulk‐density Hounsfield unit (HU) assignment algorithms are often lacking and deep learning‐based methods are instead required for robust image generation.^23^, ^24^

Errors can arise in sCT images because the process involves creation of a pseudo image set based on training data.^25^ sCT image generation has been identified as a major cause of errors in failure mode and error analysis (FMEA) studies of MRI‐only processes.^26^, ^27^, ^28^ Errors in sCT images could contribute to dosimetric differences for planning target volumes (PTV) and organs at risk (OAR).^29^ Incorrect assignment of bone and air voxels in sCT images is a reported error that could potentially lead to incorrect dose calculation and organ localization.^13^, ^15^, ^30^, ^31^, ^32^ Palmér et al. evaluated digitally reconstructed radiograph (DRR) accuracy from H&N sCT images produced via a convolutional neural network (CNN)‐based algorithm for a cohort of 14 H&N cancer patients. They noted the sCT algorithm produced misclassified bone voxels around the spinous processes.^13^ Lerner et al. reported on the presence of abnormal bone structures in a CNN‐based brain sCT image, possibly due to deviations from the characteristics in training data set.^31^ Klages et al. reported on the presence of misclassified air cavities near bone interfaces in generative adversarial network (GAN)‐based H&N sCT images.^30^ For MRI‐only radiation therapy to be implemented clinically understanding or mitigating these errors may be necessary

While bone and air misassignments have been identified in sCT images, their impact on CT number assignment to OAR structures and dosimetry has not been well characterized. This is especially important for H&N sites, which contain many interfaces between air, bone, and/or tissue and where voxel misassignments could result in appreciable dose differences. Furthermore, there have been limited studies reporting on the performance of sCTs for H&N RT plans, which tend to be very highly modulated.^13^, ^33^, ^34^, ^35^ Characterizing the effect of voxel misassignments in highly modulated treatment plans developed on sCT could allow for the development of QA criteria for the clinical implementation of H&N MRI‐only radiotherapy.

The aim of this study was to investigate if HU voxel misassignments from sCT images result in dosimetric errors in clinical treatment plans. In this work, we characterized the HU differences between paired sCT and CT images of H&N cancer patients and studied the resultant dosimetric impact. We quantified tissue voxel assignment differences by creating CT‐derived HU threshold contours for 5 tissue types and compared CT numbers across CT and sCT. Clinical IMRT plans created on CT images were recalculated onto corresponding sCT images and the dosimetric impact from differences in tissue CT numbers was quantified.

## 2 METHODS

### 2.1 Patient and imaging information

In this retrospective study, patients with H&N cancer undergoing same day CT and 3T MRI at a single institution between January 2023 – April 2023 were identified. Patient diagnosis and prescription information is presented in Table 1. Fourteen patients who received H&N radiotherapy met these criteria. All patients had CT simulation imaging on a Siemens Somatom Definition AS (Siemens, Erlangen, Germany) for clinical target volume (CTV) delineation and treatment planning. Patients were simulated using an immobilization mask for reproducible treatment setup (Integrated Shim™ for Portrait™ S‐frame immobilization masks, QFix, Avondale, Pennsylvania, USA). CT simulation images were acquired at 120kVp and 100−150 mA tube current. Images were reconstructed with a Bf37 kernel with a 1.2 mm x 1.2 mm in plane resolution and 3 mm slice thickness. Each patient had CTV and organ‐at‐risk (OAR) contours delineated on their CT simulation images by two attending radiation oncologists with specialized H&N cancer expertise.

**TABLE 1:** Summary table patient diagnosis, PTV structures, doses, and volumes.

	Diagnosis	PTV volumes (cc). Volumes denoted in parenthesis. PTV names denoted by PTV_XXXX where XXXX is the prescription dose in cGy
Patient 1	Malignant neoplasm of nasal cavity	PTV_6000 (110.34) PTV_5400 (150.27)
Patient 2	Malignant neoplasm of tonsil	PTV_6600 (204.97) PTV_5940 (433.69)
Patient 3	Squamous cell carcinoma of skin	PTV_6600 (58.99)
Patient 4	Malignant neoplasm of larynx	PTV_6600 (51.82) PTV_6000 (200.33) PTV_5600 (338.64)
Patient 5	Malignant neoplasm of tongue	PTV_6600 (153.42) PTV_5940 (380.25) PTV_5412 (197.10)
Patient 6	Malignant neoplasm of mouth	PTV_6996 (152.38) PTV_5940 (435.06) PTV_5412 (33.60)
Patient 7	Malignant neoplasm of base of tongue	PTV_6996 (60.98) PTV_5940 (240.13) PTV_5412 (201.59)
Patient 8	Malignant neoplasm of base of tongue	PTV_6996 (98.3) PTV_5940 (215.04) PTV_5412 (126.79)
Patient 9	Malignant neoplasm of mouth	PTV_6996 (27.27) PTV_6600 (38.66) PTV_5940 (646.72)
Patient 10	Malignant neoplasm of tonsil	PTV_6996 (302.55) PTV_5940 (262.39) PTV_5412 (118.21)
Patient 11	Malignant neoplasm of tonsil	PTV_6996 (149.28) PTV_5940 (187.97) PTV_5412 (130.96)
Patient 12	Malignant neoplasm of cheek	PTV_6600 (27.93) PTV_5940 (143.79)
Patient 13	Malignant neoplasm of nasopharynx	PTV_6996 (207.55) PTV_5940 (600.02) PTV_5412 (12.47)
Patient 14	Malignant neoplasm of parotid	PTV_6600 (12.48) PTV_5940 (70.99)

Same day MRI simulation images were acquired on a 3.0T Siemens Vida (Siemens, Erlangen, Germany). Patients had identical immobilization as used for CT simulation but excluding the bite block as the material was not MRI‐safe (patients were instructed to open their mouths inside the mask without the bite block being present). An UltraFlex 18 coil (Siemens, Erlangen, Germany) was suspended from the coil bridge anteriorly and brought as close to the patient surface as possible without touching the mask. Velcro straps were used laterally to bring the UltraFlex coil close to the patient's sides. The spine coil was used for posterior signal. For sCT generation, T1‐weighted Volumetric Interpolated Breath‐hold Examination (VIBE) MRI sequence images were acquired for each patient. The T1‐VIBE sequence utilizes the Dixon technique to acquire an in‐phase (water + fat), opposed‐phase (water—fat), fat only (in‐phase—opposed‐phase), and water only (in‐phase + opposed‐phase) MRI. Dotarem (Guerbet, France) contrast was used with a gradient recalled scanning sequence. T1‐VIBE MR images were acquired using an echo train length of 2, flip angle of 12 degrees, echo time of 2.46 ms, and repetition time of 5.54 ms. All images had 3D distortion correction applied. Images were reconstructed with a slice thickness of 3.2–3.5 mm and in‐plane resolution of 0.84375–0.875 mm.

SCTs were generated from T1‐VIBE Dixon MRIs using a commercially available deep learning‐based generator MRI Planner version 2.2 (Spectronic Medical AB, Helsingborg, Sweden). To mitigate any organ motion between MRI and CT simulation, the in‐phase T1‐VIBE Dixon MRI was deformably registered to the planning CT image using MIM version 7.1.4 (MIM Software Inc, Beachwood, Ohio, USA). Each MRI was resampled to the same voxel size as CT. A chained registration using the same deformable vector field was applied to out‐phase, fat and water Dixon images. The deformed MR image was visually inspected by an expert medical physicist with 6 years of experience to check if there was good agreement between the deformed MRI anatomy to the reference CT. The check included checking that bone structures and tissue interfaces such as air/soft tissue were in general agreement between the MRI and CT. The deformed MR images were used to generate sCT images.

### 2.2 HU classification evaluation

**FIGURE 1:**
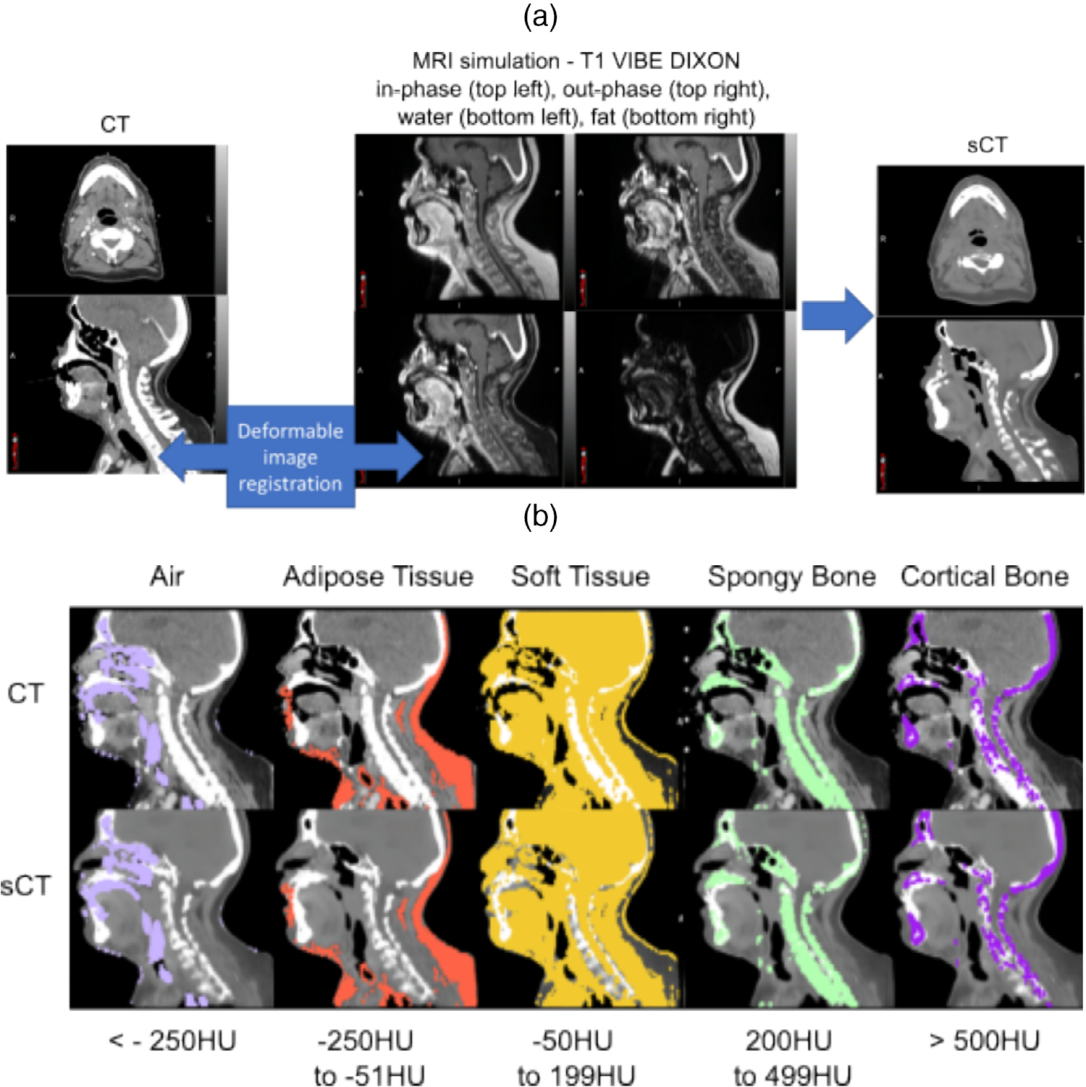
(a) Same day CT and MRI images were acquired. Deep‐learning based sCT images were generated using chain deformed T1‐VIBE Dixon MRI images. (b) Soft tissue (yellow), adipose tissue (orange), air (lilac), spongy (green), and cortical bone (purple) were segmented on CT and sCT allowing for quantification of HU differences.

Tissue voxel assignment was quantified by creating a CT‐derived HU threshold contour in MIM (Figure 1). Five tissue types based on CT number (CTn) were quantified: air (contained inside the body surface) (CTn < = −251 HU), adipose tissue (−250 HU < = CTn < = −51 HU), soft tissue (−50 HU < = CTn < = 199 HU), spongy bone (200 HU < = CTn < = 499 HU) and cortical bone (CTn > = 500 HU). These tissue types were defined for both CT and sCT images. The mean HU differences for these tissue types were recorded and T‐test statistics were calculated to determine if sCT/CT HU differences were significant (Table 3). The frequency of target volume and OAR structures that had a HU difference > = 80 HU (the CT window‐width setting for intra‐cranial structures) was computed to establish structure classification accuracy.

### 2.3 sCT dosimetric plan recomputation evaluation

Clinical IMRT treatment plans created on simulation CTs were recalculated onto corresponding sCTs to evaluate the dosimetric impact of any differences in CT number that were observed. Each patient had a 6MV photon Volumetric Modulated Arc Therapy (VMAT) plan created in Raystation version 11 (Raysearch Laboratories, Sweden), which had been optimized and approved for actual delivery to the patient. Each plan was created for delivery on a Varian Truebeam (Varian Medical Systems, Palo Alto, California, USA). The same CT to electron‐density table was used for dose calculation in both image sets. The choice of PTV doses was based on specific clinical considerations for each patient. PTV doses ranged from 5400 to 6996 cGy over 30 to 33 fractions. Each plan was delivered with a dose rate of 600MU/min. Plans included 2−3 simultaneous integrated boost (SIB) target volumes with a mean modulation factor of 3.8 ± 1.3 MU/cGy.^39^ The modulation factor was calculated by dividing the total MU delivered by the total dose delivered. A summary table of the modulation factors for each patient is included in Table 2.

**TABLE 2:** Summary table of plan parameters for each patient. The monitor units/fraction (MU/Fx), modulation factor (MF), number of arcs and prescription information is provided.

Patient number	MU/Fx	MF (MU/cGy/Fx)	#Arcs
1	1108.49	5.54	3
2	738.14	3.69	2
3	599.24	2.83	2
4	849.9	3.86	3
5	753.17	3.77	2
6	871.77	4.11	3
7	724.57	3.42	2
8	1074.49	5.07	3
9	812.77	3.83	3
10	1143.54	5.39	4
11	1097.77	5.18	4
12	321.15	1.61	2
13	796.6	3.76	5
14	247.49	1.24	2

## 3 RESULTS

### 3.1 HU classification results

**FIGURE 2:**
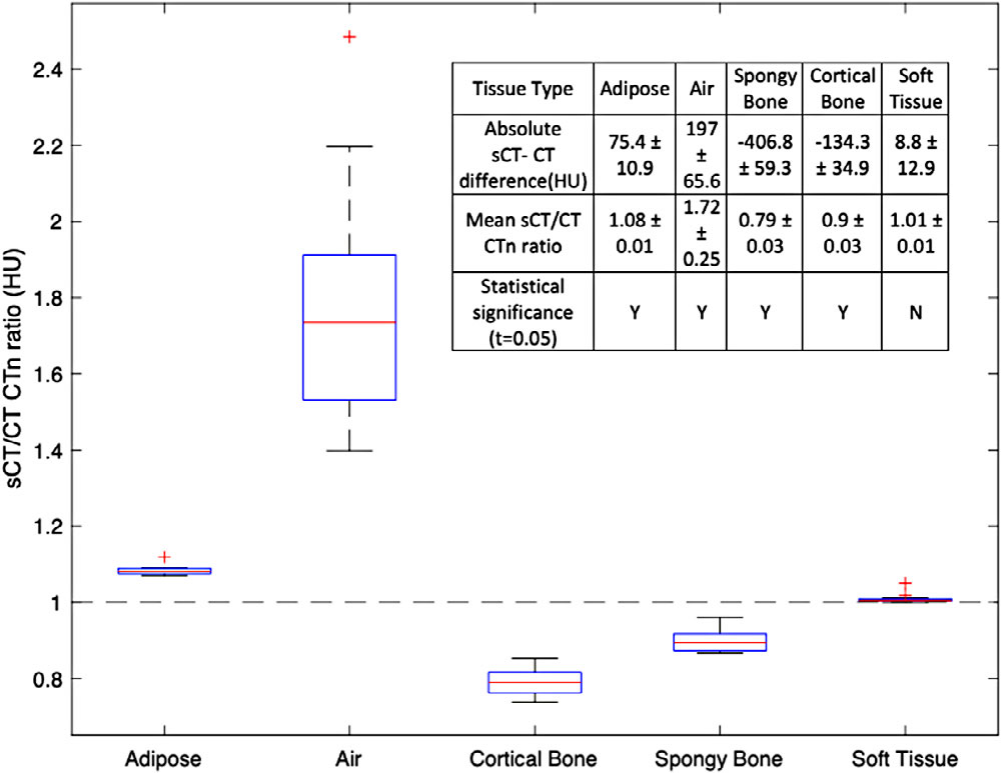
Relative and absolute sCT/CT HU differences for adipose tissue, soft tissue, air, spongy and cortical bone. The mean absolute differences and ratios between sCT/CT CT number values are indicated in the figure sub‐table for each tissue type. The sCT/CT HU significance (*t* = 0.05) is reported for each tissue type.

Figure 2 shows the relative and absolute sCT/CT HU differences for the tested cohort. t‐test statistics were calculated with a significance level of 0.05 to determine if the difference in sCT/CT CT numbers were statistically significant. The CT numbers assigned for air (contained within the skin surface) and bone showed statistically significant differences between sCT and CT. The CT/sCT agreement for soft tissue was within 10 HU for all tested patients.

**FIGURE 3:**
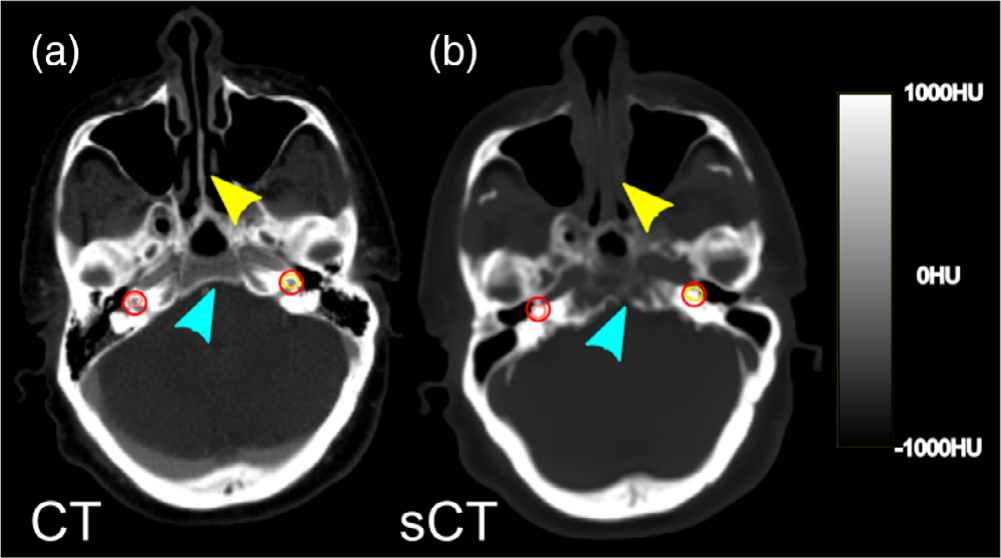
Example of tissue misclassification for the cochleae (red circle) in CT (a) and sCT (b). The cochleae are small volume structures that sit near bone, air and soft tissue interfaces and had HU differences > = 80 HU for 79% (left cochlea) and 85% (right cochlea) of the tested patients. An example of soft tissue defined in air is indicated with the yellow arrow and soft tissue as bone by the blue arrow.

Table 3 lists the five structures that contained an average CT number difference of > = 80 HU on sCT relative to CT. The most commonly misclassified structures were the cochleae, which are small (∼0.5 cc) soft tissue structures located within cortical bone/air interfaces, as shown in Figure 3. The small size and proximity to bone/air interfaces of the cochleae and temporomandibular joints make both structures susceptible to HU disagreements between sCT and CT datasets. Finally, the epiglottis, a small, mobile flap of cartilage superior to the larynx, also saw disagreement between sCT and CT scans in about 40% of cases evaluated due to its small size and mobility. All of the GTV/CTV structures had < 80 HU difference between the CT/sCT images.

**TABLE 3:** Frequency of anatomical structure misclassification in sCT. The five most common structures are indicated in this table.

Structure	Frequency of misclassification (number)	Frequency of misclassification (%)
L/R Cochlea	11/12	79%/85%
Mandible	11	79%
Oral Cavity	8	57%
Epiglottis	6	43%
L/R TM Joint	4/5	29%/36%

### 3.2 sCT dosimetric plan recomputation results

**FIGURE 4:**
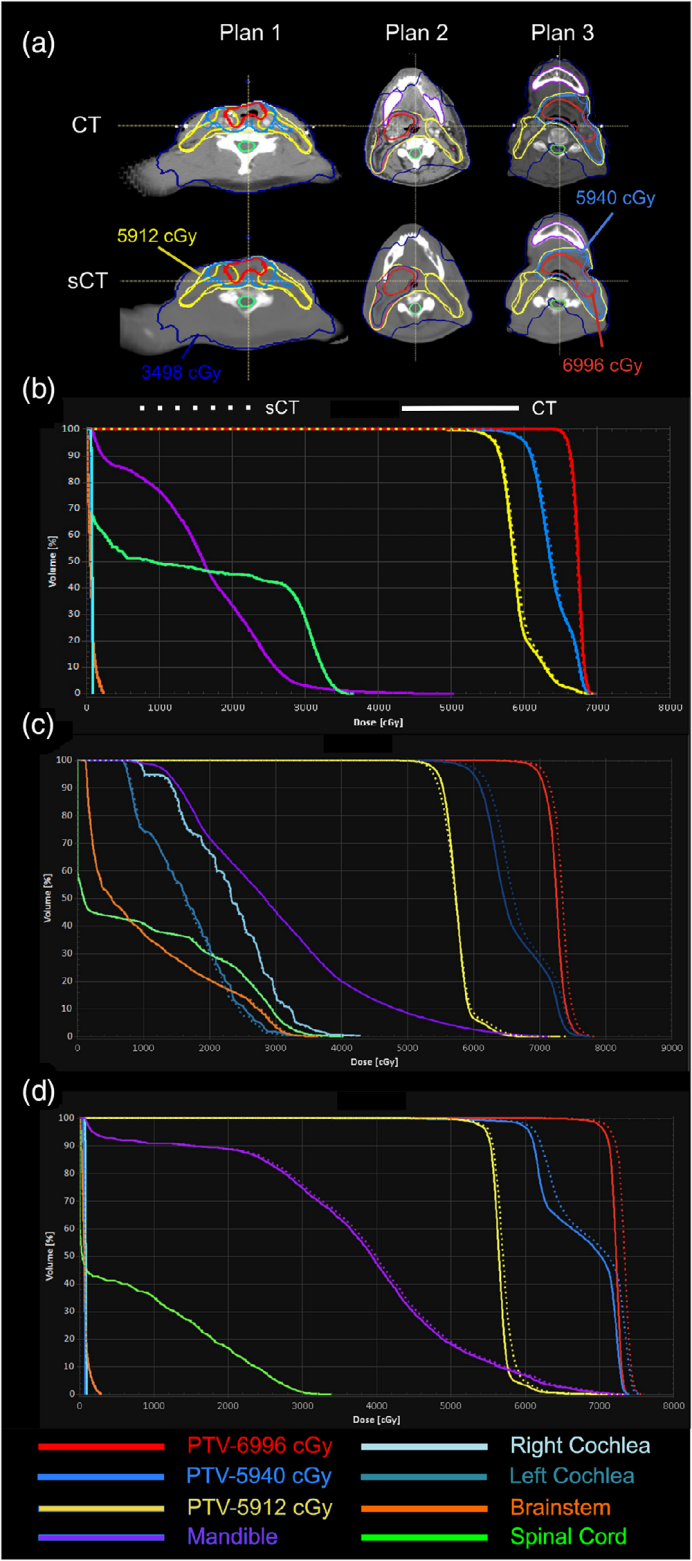
(a) Axial slice showing a CT computed treatment plan recalculated on sCT for plans with a modulation factor = 3 (Plan 1), modulation factor = 4 (Plan 2) and modulation factor = 5 (Plan 3). The D20 (720 cGy), 5412 cGy, 5940 cGy, and 6996 cGy isodose lines are shown (dark blue, yellow, light blue, red) overlaid with the 5412 cGy (yellow), 5940 cGy (light blue) and 6996 cGy (red) PTV structures. DVH for CT (solid lines) and sCT calculated plans (dashed lines) are shown for Plan 1 (b), Plan 2 (c), and Plan 3 (d).

The mean percentage differences in D95, D50 and mean doses for plans calculated in sCT and CT are shown in Table 4. The percentage differences are calculated based on: (HU(sCT)—HU(CT))/HU(CT). All analyzed plans had either 2 or 3 simultaneous integrated boost (SIB) volumes and the mean values are indicated accordingly. The percentage differences for four OAR structures are also shown. Across all plans, the largest mean percentage difference in D95, Dmean and D50 for brainstem, spinal cord, parotids and PTV doses was 1.7%. For the structures with a HU difference > 80 HU, the largest mean percentage difference in D95, Dmean and D50 was 2.3%. We observed that for 5 patients in the study that the difference in ΔD95 for the prescription PTV dose was −1.2 % ± 1.2%. For 9 patients in this study the difference in ΔD95 for the prescription PTV dose was 1.5 % ± 1.1%. We noted that as the IMRT plans became more complex (i.e., higher modulation factor), there was up to a 3% difference in the high dose PTV calculations especially for plans with modulation factors > 4 (Figure 4). The mean gamma passing rates for all plans were 95.5% ± 2.0% (3%, 3 mm, 10% threshold, global) and 92.7% ± 2.1% (2%, 2 mm, 10% threshold, local).

**TABLE 4:** Percentage differences in D95, D50 and mean doses for PTV and OAR structures including the structures identified with > 80 HU difference and major critical structures including the left and right parotid glands, brainstem and spinal cord for all patients. Differences are calculated based on (HU(sCT)—HU(CT))/HU(CT).

		ΔD95 (%)	ΔDmean (%)	ΔD50 (%)
Structures with HU difference > 80 HU	Cochlea L	2.3 ± 3	1.8 ± 1.9	2 ± 2.1
	Cochlea R	1.3 ± 1.9	0.8 ± 1.7	0.7 ± 2.1
	Mandible	1.1 ± 1.9	0.2 ± 1.9	0.2 ± 2.1
	Oral Cavity	1.5 ± 3.8	0.4 ± 1.2	0.8 ± 1.6
	Epiglottis	0.7 ± 4.5	0.3 ± 3.5	−0.3 ± 4.1
	Joint TM L	0.2 ± 1.6	0.3 ± 0.9	0.4 ± 1.5
	Joint TM R	1.1 ± 0.9	0.4 ± 0.8	0.4 ± 1.1
Sample Plan Metrics	Brainstem	−0.4 ± 2.7	0.8 ± 1.7	1.1 ± 2.1
	Spinal Cord	1.7 ± 3.3	0.3 ± 1.2	−0.8 ± 1.8
	Parotid L	0.4 ± 0.9	1 ± 1.4	0.6 ± 1.3
	Parotid R	0.2 ± 1	0.3 ± 1.4	0.1 ± 1.1
	PTV 1 (prescription)	0.5 ± 1.8	0.7 ± 1.9	0.4 ± 1.7
	PTV 2 (if present)	0.4 ± 1.4	0.5 ± 1.6	0.6 ± 1.7
	PTV 3 (if present)	0 ± 1	0.2 ± 1.4	0.2 ± 1.5

## 4 DISCUSSION

In this study we investigated the differences between CT number assignment in sCT and CT images and the types of clinical structures that are most impacted by HU differences. We investigated the dosimetric impact of HU assignment differences between sCT and CT images in a cohort of highly modulated clinical H&N IMRT treatment plans. This study demonstrated there were statistically significant differences in HU assignment for bone and air. However, those differences in HU assignment seemed to have little impact on OAR structure doses.

The dosimetric investigation performed in this study demonstrated that there seemed to be little effect of HU misassignments for OARs particularly with attention to small structures such as the cochleae, epiglottis, and temporomandibular joints. We noted for 3 patients that there was some deviation between the sCT and CT doses as the plan complexity increased. This was noted since more complex volumetric modulated arc therapy (VMAT) plans may have some increased sensitivity to variations in tissue/HU assignment because more complex beamlet shapes are impinging through heterogeneous anatomy. Since, beamlets in H&N plans can pass through a bone/air, bone/tissue, and tissue air interfaces, further investigations into the effect of sCT errors are warranted. Further studies on the dosimetric effect of plan complexity with sCT HU misassignments is warranted.

Other studies of deep‐learning‐based sCT methods for the H&N have also showed differences in mean tissue classification errors between bone and air but good HU agreement with soft tissues. Palmér et al evaluated the geometrical and dosimetric differences between CT and H&N sCT images and showed a mean error (± 1 standard deviation, sd) of −1 ± 7 HU for soft tissue, −62 ± 28 HU for bone, and 107 ± 75 HU for air. The mean MAE was 67 ± 14 HU for overall body, 38 ± 6 HU for soft tissue, 195 ± 27 HU for bone, and 198 ± 68 HU for air. This study reported a mean gamma pass rates ranged from 95.7% to 99.9%, in good agreement with the findings from prior studies.^15^ However, this study additionally showed that there could be tissue‐specific HU misassignments in bone/air interface regions which may need to be taken into consideration when reviewing H&N MRI‐only plans.

Limitations of this study include the impact of deformable image registration on the accuracy of sCT generation and the generalizability of the findings. To compare sCT generation accuracy we wanted to ensure that the MRI anatomy agreed well with CT anatomy. Hence all patients in this study had same day MRI/CT simulation imaging, typically sequentially, using close‐to‐identical patient positioning. However, since the MRI and CT simulation machines are not in the same room patients were physically moved from the CT simulation session to the MRI simulation session. Thermoplastic masks were removed between sessions and the bite block could not be used in MRI simulation. Therefore, even though most of the simulation sessions were sequential, there could have been internal anatomical motion between sessions in addition to slight difference in tongue and jaw position. To account for this, deformable image registration was used to correct any anatomical differences between MRI and CT. The deformed MRI images were manually inspected by a medical physics expert to ensure that the anatomy agreed well with CT before sCT generation.

A second limitation of this study was that only a single deep learning‐based method was tested for a single anatomic site. We specifically chose to investigate deep learning‐based methods because they have, in general, better tissue assignment accuracy and reproducibility compared to their bulk‐density counterparts.^30^, ^31^ The H&N site was specifically selected because of the complexity of the anatomy involved in sCT generation. Additionally, the highly modulated external beam radiotherapy plans used to treat H&N cancers were thought to provide the most rigorous testing of the integrity of sCT images.

Spatial inaccuracies in MRI can propagate into synthetic CTs if the parent MRI hasn't been corrected. Images from our study were corrected for geometric distortion by applying 3D distortion correction during MRI acquisition. 3D geometric distortions were quantified using a large field MR distortion phantom. Patient specific distortions can occur from a variety of sources including chemical shift differences and local susceptibility variations. Studies have demonstrated that the effects of the errors contribute up to 0.5 mm depending on the readout bandwidth.^36^ For H&N patients, susceptibility artifacts can appear prominently around dental implants.^37^, ^38^ The effect on dental susceptibility artifacts on sCT imaging has not been studied however in this study we did not note imaging or dosimetric deviations due to the presence of dental implants. However, the presence of dental implants on the sCT image generation merits further study.

The goal of this work is to report on the dosimetric impact of HU differences for OARs in H&N sCT planning. Towards this future goal, one class of studies should include investigating additional deep learning/artificial intelligence (AI) methods to evaluate structures misidentified in sCT. Improved sCT models could be developed by using additional training data and incorporating different MRI sequences such as ultrashort echo time MRI, which increases bone signal and could improve bone/air contrast and reduce bone/air sCT HU misclassifications.^40^ Additionally, further studies should include the development of QA tools and/or sCT correction methods using secondary deep learning models based on only MRI as input.

## 5 CONCLUSION

In this study we quantified differences in HU values between paired CT/ sCT of H&N cancer patients and investigated the dosimetric impact on clinical treatment plans. In this cohort of patients, HU differences in sCT were observed but this did not lead to large differences in OAR doses for multiple analyzed structures, PTV D95 metrics or gamma passing rates. Additional investigation of potential dosimetric consequences of this error could inform QA procedures using MRI‐only RT planning and lead to development of mitigation or correction strategies.

## AUTHOR CONTRIBUTIONS

**Kamal Singhrao**: Responsible for study design; data collection; dosimetry analysis; and manuscript writing. **Catherine Lu Dugan**: Responsible for data collection and curation; imaging analysis; and manuscript writing. **Christina Calvin**: Aided in MRI patient data collection and MRI protocol selection for study. **Luis Pelayo**: Aided in MRI patient data collection and MRI protocol selection for study. **Sue S. Yom**: Provided medical expertise on H&N radiotherapy; provided oversight on clinical treatment plans; and proofread manuscript. **Jason W. Chan**: Provided medical expertise on H&N radiotherapy; provided oversight on clinical treatment plans; and proofread manuscript. **Jessica Scholey**: Responsible for study design; oversight of data collection/integrity; and manuscript proof reading. **Lisa Singer**: Responsible for study design; oversight of data collection/integrity; and manuscript proof reading.

## CONFLICT OF INTEREST STATEMENT

The authors declare no conflicts of interest.
